# Radial keratotomy: background and how to manage these patients nowadays

**DOI:** 10.1186/s12886-023-03261-0

**Published:** 2024-01-04

**Authors:** Guilherme Novoa Colombo-Barboza, Pablo Felipe Rodrigues, Fernanda Daroz Paulo Colombo-Barboza, Bernardo Kaplan Moscovici, Luiz Roberto Colombo-Barboza, Marcello Novoa Colombo-Barboza, Walton Nose

**Affiliations:** 1 Department of Ophthalmology, Hospital Oftalmológico Visão Laser, São Paulo, Santos Brazil; 2 Department of Ophthalmology and Visual Sciences, Federal University of São Paulo (UNIFESP), São Paulo, Brazil; 3 Department of Ophthalmology, Santos Medical Sciences (UNILUS), Santos, Brazil; 4 Department of Ophthalmology, UNIMES Medicine College, Santos, Brazil

**Keywords:** Radial keratotomy, Refractive surgery, Cornea, Corneal surgery

## Abstract

In this review, we presented the principles of radial keratotomy (RK), its evolution, enhancement, and complications, and strategies to manage the consequences of RK in the present day. It is essential to understand the RK procedure f, the theoretical background that supported this surgery, the current effect on the cornea, and how to approach patients needing vision improvement. These patients are developing cataracts that need to be handled well, from the IOL calculation to the surgical procedure. Guided keratorefractive surgery is the most accurate procedure to improve these patient's vision and life. Nevertheless, some patients may need other approaches, such as sutures, penetrating keratoplasty, corneal rings, and pinhole implants, depending on the degree of irregularity of the cornea, ablation depth for guided surgery or if the sutures are open.

## Background

Leendert Jan Lans, a Dutch ophthalmologist, conducted the first systematic laboratory research on refractive surgery in rabbits, defined the principles of radial keratotomy (RK), and was the first person to describe the *coupling phenomenon* [1–7].

Donder's Treatise on Eye Accommodation and Refraction Anomalies has effectively addressed the clinical and optical principles underlying refractive errors. This knowledge enabled future clinicians to develop surgical techniques to correct astigmatism and myopia. In 1885, Schiot*z*became the first ophthalmologist to treat astigmatism via incisions [8]. Four months following the cataract surgery, he conducted a limbic incision to reduce astigmatism of 19.50 diopters (D) to 7.00 D. After studying six patients with traumatic and surgical peripheral corneal scars who developed corneal flattening on the meridian that crossed the scar without any alteration in the perpendicular meridian, Bates proposed an operation for astigmatism in 1894. The Dutch ophthalmologist Faber was the first to perform anterior transverse keratotomy to improve his patients’ vision and enable them to meet their occupational needs [3, 9, 10]. In 1896, Lucciola reported 10 cases of nonperforating corneal incisions to flatten the steep meridian [11]. Lans' doctoral thesis, “Experimental studies of the treatment of astigmatism with non-perforating corneal incisions,” was the first published description of systematic trials documenting the effects of nonperforating corneal incisions in rabbits.

Lans' research yielded the following essential aspects of RK: [7].

**Fig. 1:**
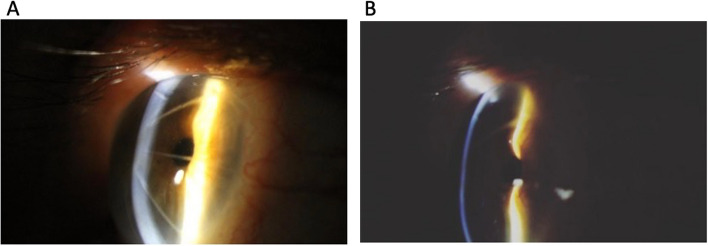
**a** Peripheral bulging and central flattening are induced by nonperforating corneal incisions perpendicular to the limbus. **b** Healing of keratotomy wounds causes additional flattening of the central cornea

- Deeper incisions exhibit a more significant impact.
- Nonperforating corneal incisions perpendicular to the limbus cause peripheral bulging and central flattening (Fig. 1a).
- Keratotomy-associated wound healing induces further flattening of the central cornea (Fig. 1b).
- Radial wounds flatten the central cornea and create a peripheral inclination in the meridian parallel to the wounds (Fig. 1b).

Sato pioneered refractive surgery in the current models for myopia. He observed corneal flattening in keratoconus patients with ruptured Descemet membrane and subsequent hydrops. Subsequently, he designed the “Sato scalpel,” a modified Lapersonnés scalpel with a sharp-angled tip capable of entering the anterior chamber through the limbus.

Sato performed posterior keratotomy in ten eyes of eight patients with keratoconus in 1939 and documented corneal flattening [12]. Hundreds of patients underwent anterior and posterior keratotomy for myopia in the early 1950s. While the surgeon proceeded from table to table, the patients were queued up at tables adjacent to each other. If an aqueous leak was discovered, the surgeon would stop, operate on the next patient on the adjacent table, and then return to the first patient to complete the operation. Sato et al. published their experience with anterior and posterior incisions in 32 eyes in 1953. They concluded that “this novel surgical procedure is a proven and safe method that definitively cures or satisfactorily relieves more than 95% of all cases of myopia in Japan.” [13].

Unfortunately, the significance of the corneal endothelium was not fully understood at the time. The first occurrence of corneal decompensation in a patient undergoing “Sato surgery” was not documented until 15 years after Sato’s death. Despite the tragic consequences of Sato's work, his data regarding rabbits and clinical findings supported Lans' findings and established the following: the effect of keratotomy increases with greater depth, number, and length of the incision; radial incisions placed in a meridian flattened the meridian as well as the perpendicular meridian slightly; and crossed incisions produced more pronounced scars and should be avoided. Sato's experiments with rabbits did not provide information regarding the significance of endothelium because rabbit endothelium possesses regenerative capacity.

In 1960, shortly after Professor Sato's death, Fyodorov traveled to Japan, where he met Akiyama and learned Sato's RK technique. After learning Yanaleyev's preliminary results in 1969, Fyodorov and colleagues decided to reapply RK and establish mathematical formulas based on anatomical and mechanical parameters of the cornea to determine the most precise outcomes for each patient. He discovered that optical zones < 3 mm reduced visual acuity [14]. Fyodorov emphasized the significance of establishing an individual coefficient to accommodate for variances in surgical technique, particularly regarding incision depth. In 1974, Fyodorov started practicing early RK on patients using free-hand razor fragments in a blade holder. A depth meter was used to measure the incision depth. In 1978, crystal blades were used, and protected knives were manufactured in 1979. Fyodorov observed that the central cornea flattened, and the peripheral cornea became steeper following surgery using videokeratoscopy. He explained it as intraocular pressure (IOP) pushing the cornea forward after rupturing the deep circular ligament [15]. Fyodorov reported exceptional results: 1.5 years following surgery, all 230 eyes with previous refractive errors of − 1 to − 6 D achieved final refraction within 0.50 D of emmetropia [16]. These exceptional results, however, have never been replicated.

Bores performed the first RK in the United States in November 1978 at the Kresge Eye Institute. Bores, Myers, and Cowden published the first American results in 1981 after studying 303 eyes that had undergone surgery with 16 previous radial incisions and observed that one year following the treatment, 65% of eyes achieved uncorrected distance visual acuity (UDVA) better than or equivalent to 20/40 [17]. Bores began the funded project *Prospective Evaluation of RK*(PERK) in early 1981, which examined the performance of eight centrifugal incisions, with the diameter of the optical zone determined by the spherical equivalent of refractive error. The effect of age on the outcome was not investigated at this time. The PERK study discovered that 17% of patients experienced hypercorrection of > 1.0 D after five years of surgery. Between 6 months and 10 years following surgery, 43% of eyes exhibited hypermetropic deviation ≥ 1.00 D, with an annual increase of 5%. The progression of approximately + 0.06 D annually was also observed between 2 and 10 years following surgery without a tendency to stabilize [1, 18, 19].

However, various legal hurdles prevented the American ophthalmologic community's early practical implementation of RK. Although RK was well-received, it did not gain popularity until the mid-1980s. However, with the publication of PERK data and other studies that aimed to clarify the safety, efficacy, predictability, and stability of the RK technique, there was tremendous interest in adopting the treatment.

O'Dell and Wyzinski described a series of bilateral RKs in 27 adolescents. On average, 22 months following surgery (3–54 months), 64% of eyes attained emmetropia. Although patient satisfaction was high, the research highlighted the issues of hypercorrection-induced astigmatism, the need to wear glasses following surgery, and repeated reoperations to cope with increased myopia as the patients aged. RK was thus discouraged in patients aged < 21 years. However, RK had no upper age limit.

The aim of this review is to explain the theoretical background behind this surgery, its complications and how to deal with them.

### Were the refractive parameters for considering keratorefractive surgery different?

Refractive correction following RK was determined according to the patient's age, the diameter of the selected optical zone, the number of incisions, and depth. These variables were related to each patient’s desired refractive surgical effect.

The predictability obtained in the surgery ranged from − 1.5 to − 2.0 D in 80%–90% of cases [20–22]. Patients interested in refractive surgery could be unwilling to accept this “unpredictability”. When compared with intrastromal rings, the predictability of RK was superior in high myopia.

In the early 1980s, it was established that the patient’s age substantially impacted the surgical outcome. The PERK study’s regression analysis documented an effect of 0.40–0.60 D higher per decade [22, 23]. Additionally, when the optical zone shrank, the variability of the refractive effect increased [22].

### Importance of the diameter of the optical zone

Fyodorov and Durnev were the first to propose that decreasing the diameter of the optical zone would more considerably reduce myopia [15]. Later, Salz et al. demonstrated a statistically significant effect in human cadavers by lowering the diameter of the optical zone between four and eight incisions.

The PERK study documented a reduced optical zone diameter from 4.0 to 3.5 mm associated with an increasing refractive change of 0.68 D, and a reduction in optical zone diameter from 3.5 to 3.0 mm associated with an increasing refractive change of 1.08 D [22].

Although optical zones of < 3.0 mm will boost the effectiveness of the surgery, they will most likely cause more significant glare.

### Assessment of the number of incisions

The number of incisions used for RK has decreased over time [15]. In 1980, Schacher devised a theoretical corneal model; his work was validated by a clinical study comparing 8 and 16 incisions utilizing an optical zone of 3 mm that achieved a reduction in myopia of 5.21 and 5.18 D, respectively [24–26].

Several studies have broadened the search for optimal incisions in mild and moderate myopia [1–25]. Clinical trials have demonstrated that a four-incision RK effectively corrects low-to-moderate myopia, with few patients being hypercorrected. According to the PERK study, in which an eight-incision surgery was employed, 94% of patients with myopia (− 2.00 D to − 3.12 D) had a UCVA of 20/40 or better, and they have residual refractive error > 1D [27]. Salz hypercorrected 21% patients, and Spieglernan reported that 68% and 84% patients with moderate myopia (− 3.25 D to − 4.37 D, respectively) had UCVA of 20/40 or better, and no patient was hypercorrected by > 1 D [28, 29]. Following the application of four further incisions, 49 (93%) patients in the Salis series had 20/40 or better UCVA, and 96% were within 1 D of hyperopia. In terms of intermediate myopia (− 3.25 D to − 4.37D), the PERK study found that 79% of patients had 20/40 or better UCVA, whereas 20% were hypercorrected by > 1 D [26–67].

Thorton presented a nomogram that considered age, sex, intraocular pressure(IOP), corneal thickness and diameters, and keratometry in his recommendation for myopia therapy, assuming that the incisions reached up to 90% of corneal depth. The correlation between myopic power change and the number of incisions/optical zone is shown in Table 1. Nordan proposed a nomogram to treat corneal toxicity (Table 2), and Casebber proposed a nomogram for hypermetropic treatment (Table 3).

**Table 1:** Nomogramproposed by Thorton for performing R.K. in a patient with exclusive myopic component

PROPOSAL TO CHANGE MYOPIC POWER	OPTICAL ZONE (NUMBER OF INCIÕES REQUIRED IN PARENTHESES)
0.75—1.12	5.00 (8) 4.75 (4)
1.13—1.49	4.75 (8) 4.50 (4)
2.12—2.61	4.25 (8) 4.00 (4)
2.62—3.11	4.00 (8) 3.75 (4)
3.12—3.73	3.75 (8) 3.50 (4)
3.75—4.36	3.50 (8) 3.25 (4)
4.37—5.11	3.25 (8) 3.00 (4)
5.12—6.11	3.00 (8)
6.12—7.50	3.00 (8 and reset to 98% 5 mm optical zone)
7.51—8.00 (OR MORE)	3.00 (8 and reset to 98% 5 and 7 mm optical zone)

**Table 2:** Nomogram proposed by Nordan for the treatment of astigmatism

ASTIGMATISM (D)	OPTICAL ZONE	ARC LENGTH (MM)	NO INCISION	NO RADIAL INCISION
1.00—1.50	7.0	4.5	1	1
1.75—2.25	7.0	3.5	2	2
2.50—4.00	7.00	4.5	2	2

**Table 3:** Nomogram proposed by Casebeer for hypermetropic treatment

CORRECTION (D)	OPTICAL ZONE	CROSS-SECTION LENGTH
1.00	6.00	2.5
1.25	6.00	3.0
1.50	5.75	2.5
1.75	5.75	2.5
2.00	5.50	2.5
2.25	5.50	3.0
2.75	5.25	3.00

### Direction of incision

RK incisions were conducted in three different ways:

1. Centrifugally: from the optical zone to the limbus; the most commonly performed procedure owing to the lower danger of entering the optic zone, which could result in meaningful glare, irregular astigmatism, corneal scarring, and loss of the best corrected visual acuity
2. Centripetally: from the limbus to the optical zone
3. Two passages: optical zone to the limbus and from limbus to the optical zone

Previous research revealed that centripetal incisions were more superficial than centrifugal incisions; hence, the former would be less effective for myopia treatment [30, 31]. The two-pass surgery gave the surgeon the benefits of centripetal surgery while maintaining the safety of the centrifugal procedure.

## Prevention and management of complications associated with radial keratotomy

Although severe consequences of incisional keratotomies have been observed and are frequently described as preventable, ophthalmologists must become acquainted with them as they will encounter patients who have undergone this procedure. Early recognition and immediate therapy may reduce poor results in the case of a complication.

### Intraoperative complications

Intraoperative complications are listed and explained in Table 4.

**Table 4:** – RK Intraoperative complications

*Inaccurate visual axis marking*	Inaccurate marking of the visual axis may result in incisions that penetrate the optic zone, causing glare, substantial irregular astigmatism, and even monocular diplopia
*Intersection of incisions*	Patients with these symptoms frequently have recurring, open corneal wounds that are difficult to heal. A surgical treatment plan will be described later in the chapter
*Microperforations*	Aqueous loss is minimal in microperforations. There is no shallowing of the anterior chamber, and the procedure may be continued at the surgeon’s discretion Initially, the microperforation incidence ranged from 0.006% to 35% [32, 33]. More recent reports employing an ultrasonic pachymeter indicate that the incidence increased from 2 to 10% following calibration [34]. Microperforations occurred more often in the inferotemporal corneal region because these regions are typically the thinnest [35].
*Decentralization*	The smaller the clear zone, the more the decentralized effect, with increased obfuscation and occurrence of irregular astigmatism. Other possible intraoperative issues included incisions along the visual axis, the wrong number of incisions, and incisions that crossed the limbus, which increased the risk of corneal neovascularization, especially if the patient used gelatinous contact lenses
*Incisions beyond the transparent cornea*	RK incisions may extend from the optical zone of the cornea to the corneal–scleral limbus or the limbal vascular arcades. Due to the incision's concurrent vascularization, limbus-penetrating incisions can render the patient intolerant to contact lenses. Because of the concurrent vascularization of the incision, limbus-penetrating incisions can render the patient intolerant to contact lenses. Fibrovascular development may cause corneal destabilization over time, resulting in considerable diurnal variation and advancement of the refractive error
*Endothelial loss*	Late corneal decompensation after Sato's procedure was a significant issue at the onset of the American experiment. The loss of endothelial cells in the initial years following THE ranged from 3 to 10% [39]. Endothelial cell loss was more significant in eyes with microperforations than in those without. Additionally, eyes with central optical zones ranging from 3.0 to 3.5 mm exhibited a statistically significant mean cell density, cell perimeter, and lateral length shift. These characteristics did not change in eyes with central optical zones ranging from 3.75 to 4.50 mm

#### Side effects

Halos, starbursts, daily visual fluctuation, and immediate postoperative regression are self-limiting adverse effects. These side effects may last beyond the perioperative phase in a limited subset of individuals [1].

### Halos and starburst effect

Halos and starbursts can be reduced in patients with varied incisions by minimizing the selection of small optical zones. Corneas with irregular astigmatism can also produce these symptoms. Irregular astigmatism and the induction of high-order aberrations (HOA) correlate with the optical zone and number, quality, and depth of incisions as well as arcuate incisions. Smaller optical zones are correlated with more HOA induction and arcuate incisions, especially incisions lacking wound repair. A deeper and increased number of incisions was correlated with a higher probability of halos and glare following the surgery.

### Daytime visual fluctuation and early regression

Visual variation throughout the day during the first postoperative months is most likely associated with immature wound architecture and corneal stromal edema adjacent to radial incisions. The corneal edema worsens at night when the eyelids are closed, resulting in normal cornea flattening. The cornea gradually returns to its original thickness throughout the day as deturgescence occurs. The amplitude of daytime fluctuation would decline each day as the wounds matured and stabilized. This characteristic may be linked with hyperopia in the early morning hours and symptom relief in the afternoon.

### Postoperative complications

The list of postoperative complications is shown in Table 5.

**Table 5:** List of postoperative RK complications

Related to vision	Unrelated to vision
Hypercorrection: This is most observed in older patients [1].	Corneal scars
Progression: typically observed in incisions extending up to the limbus. The use of contact lenses following a procedure	
Hyperopia	
Regression: related to the surgical wound healing process	
Induction of astigmatism: characterized by few and/or asymmetric incisions or lack of prior planning	
Corneal melting or infection due to incision dehiscence	

### How to manage some of the postoperative complications?

#### Rigid contact lenses

The use of reverse curvature contact lenses improves visual acuity while decreasing visual complaints related to the abovementioned events. The technological advancement provided by oblate scleral lenses usually helps improving the irregular astigmatism.

### Traumatic rupture of keratotomy scars

Several laboratory investigations on wound resistance following RK have been reported [39–41]. Larson et al. discovered that the force necessary to rupture the rabbit globe 90 days after the eight-incision RK was ~ 50% of that required to rupture the eyeballs of nonoperated controls. In a porcine model, Rylander et al. demonstrated that ocular rupture occurred more frequently at the equator in normal eyes and at the corneal incisions in the post-RK eyes [42–44].

After a corneal incision of partial or total thickness, collagen fibers do not heal from end to end, covering the entire cornea, but instead deposit a new extracellular matrix that cements both sides of the incision, resulting in a corneal scar that has less tensile strength and is permanently weaker than the natural cornea [40].

To lessen the risk of trauma following RK, it was recommended that patients be informed about this possible complication and that they wear goggles during vigorous contact sports and recreational activities, such as football, basketball, karate, and racquet-based sports. This strategy becomes increasingly valid as we learn more about corneal histology following RK. Bowman layer ruptures are permanent; an epithelial buffer is generated as the basal epithelial layer. Significant variability in scars was observed in the same individual, which is associated with the realignment of collagen fibrils following RK incisions.

### Proposals for keratoplasty secondary to radial keratotomy (corneal sutures)

The prospect of RK having a continuous effect on the cornea has been mentioned in the literature, as it can cause corneal flattening, hypercorrection, and hyperopia [27, 48]. The persistent influence of radial incisions on corneal curvature reduces the predictability of surgical outcomes, thereby rendering RK less advantageous for correcting ametropia. Consequently, strategies for halting the process and fostering a more stable refractive outcome must be developed. The concept of interrupted sutures (in a combination of 4–8 sutures) produced a satisfactory model but with low predictability [49].

It might be an exciting mechanism in perfect scenarios, resulting in hyperopia. A circular suture may promote better balance and corneal symmetry, often alleviating hyperopia. Grene Lasso et al. described the suture in 1998, which was a single circular intrastromal suture. As the suture was applied in the corneal plane, the effect was lost faster [50].

**Fig. 2:**
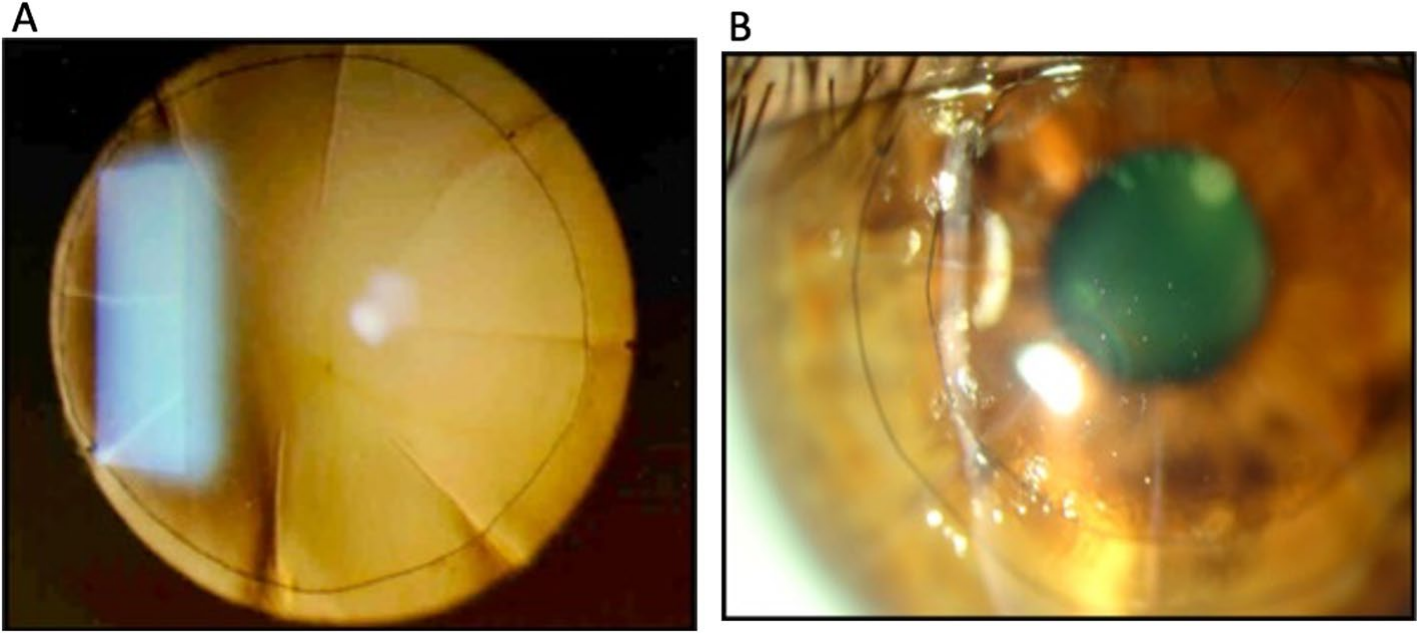
**a** Presence of more extensive radial incisions, very close to the visual axis. We observed the presence of a double circular suture. **b** Application of the circular suture favors the restoration of the prolate shape of the cornea without manipulating the optical zone

**Fig. 3:**
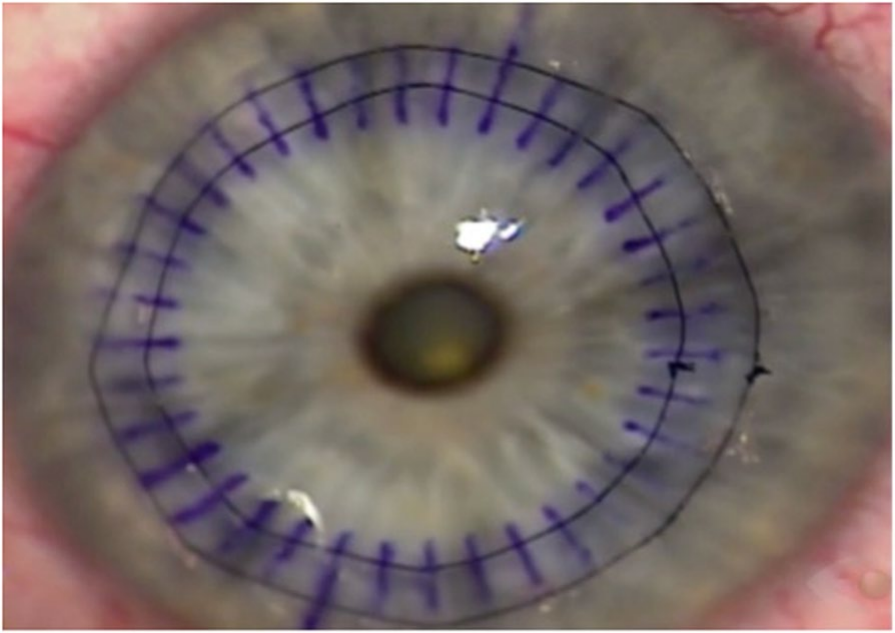
The nylon thread is passed over the incision, and the needle is inserted after the incision before the other scar; we continue applying the suture until it completes the entire turn, tightening the knot, and burying it to the corneal stroma

The double circular suture for treating hyperopia following RK, as published by Nosé et al. in 2007, used the implantation of two continuous circular sutures with nylon 9.0 in diameters of 7 and 9 mm as a procedure (Figs. 2a, 2b). The suture is initiated in the intact stroma immediately following the incision, at a depth of ~ 90%, leaving the needle before the next incision. v (Fig. 3). His research involved 17 eyes of 15 hyperopic individuals who had undergone RK and followed up for 11.6 ± 3.2 years. The corresponding spherical refraction was reduced from 4.38 ± 2.87 D to 0.54 ± 2.59 D (*P* < 0.001). The degree of astigmatism was unaffected by suture application (*P*= 0.15). Before the sutures, none of the eyes presented CDVA ≥ 20/20. After the treatment, three eyes (17.6%) attained a CDVA of 20/20, seven eyes (41.2%) experienced improvement by two or more Snellen lines, and one eye (5.9%) suffered a loss of two Snellen lines [51].

**Fig. 4:**
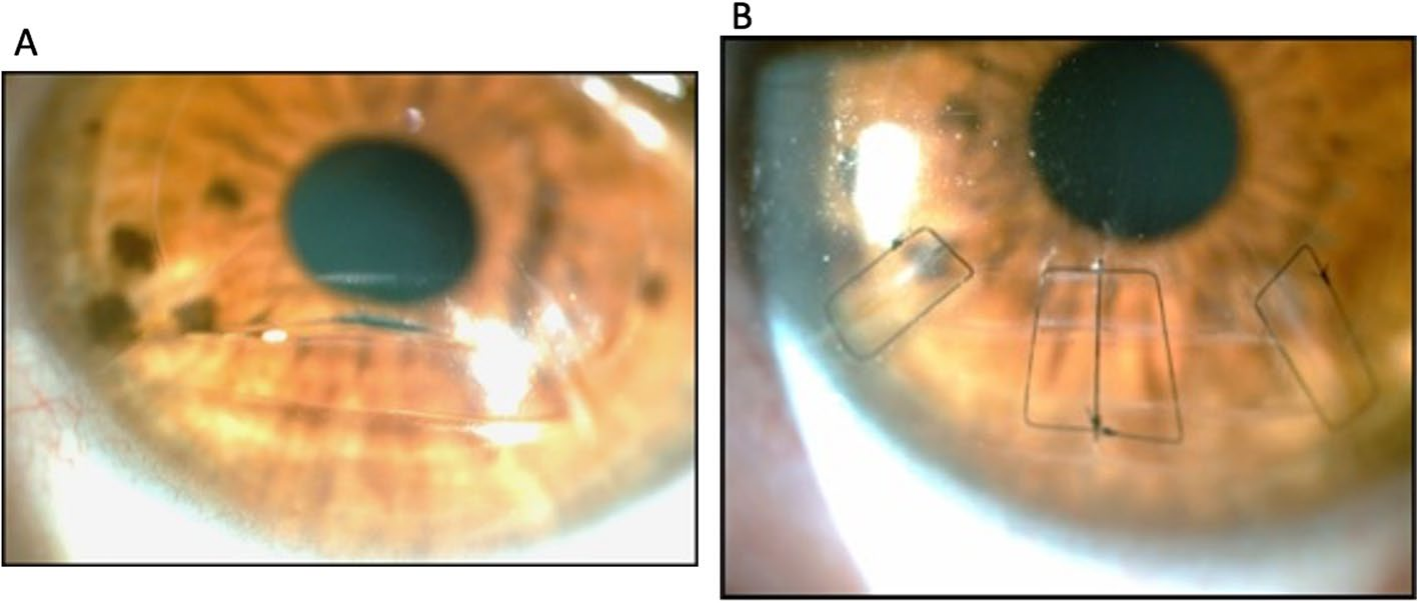
**a** Corneal transverse incision with edge exposure and noncongruence of the surgical wound. **b** Corneal sutures interrupted. Transfixation occurs at the end of the corneal incision (RK). One can observe the possibility of obtaining symmetry and parallelisms. A simple, interrupted suture is applied in the presence of large previous corneal incisions to improve the edge approach

Currently, we employ square stitches for simple sutures in one or more radial or transverse incisions. This stitch is known as Donatti's suture, modified by Nosé. The thread always goes deep into the stroma, parallel to the cut, which is an advantage of this suture. The thread passes over the incision and is sutured in a quadrangular or rectangular shape. (Figs. 4a, b). The main advantage of this suture over simple sutures is that simple sutures dissect the stroma and lose their impact rapidly, and there is no time for the wound to heal fully. Furthermore, the square suture maintains its influence for an extended time and with a more significant effect.

### PRK after RK

Guided surgery is the most secure and predictable treatment for irregular astigmatism following RK. Filev et al. performed PRK following RK in 16 eyes without problems, where the authors corrected only the refractive ametropia without correcting the HOA of the cornea that sometimes occurred following RK [52]. Topoguided or wavefront-guided procedures, primarily with PRK, have been widely employed to rectify irregular corneas (57). Motwani et al. reported successful topoguided correction of HOA using EX500 (Alcon, Fort Lauderdale, USA) [53]. Owing to the incisions, it is more practical to undertake this type of correction using PRK rather than LASIK because LASIK may have exerted an unforeseen effect on the incisions. De-epithelization must be cautiously performed to avoid damaging the incisions. The use of alcohol or a transepithelial approach can help prevent this complication. The surgeon's experience and the quality of the pictures obtained should be used to decide whether to correct HOA across the optical system (waveguided) or just the cornea (topoguided). If achieving reliable images in wavefront or topography is impossible, the guided procedure should be avoided, or another platform should be tested, this happens mostly in highly aberrated corneas. Furthermore, some patients already have lens opacity, affecting wavefront readings across the optical system, and wavefront surgery should be avoided. Conversely, wavefront treatments typically provide better refractive results as they can examine low (LOA) and HOA.

Because the correction of HOAs can generate LOAs, it is critical to analyze the ablation patterns of the treatment in topoguided (TG) treatments. In some situations, attaining acceptable postoperative results is impossible, necessitating a second surgery aimed solely at correcting the LOA. It is vital to study the ablation profile and, in most cases, choose the HOA that needs to be corrected or make some treatment modifications to achieve an improved refractive outcome when performing a topoguided correction of irregular corneas.

Finally, topoguided surgery has the disadvantage of excess tissue being removed. Vector planning (combining topographical astigmatism and refractive cylinder) was an effective mode for retreatment with excellent results with lower order aberrations and, consequently, HOAs by including both refraction and topography astigmatism in the treatment plan [64].

Mitomycin C is applied for 1 to 2 min after the excimer laser ablation to minimize corneal haze.

Guided surgery following RK presents good refractive results and improved quality of life in patients who have undergone RK, especially those with irregular astigmatism. Usually, it requires experience to perform this procedure, as it is advisable to analyze the ablation profile to avoid inducing refractive errors [54–61]. Treatment with TCAT depends exclusively on topographical maps of the cornea to remodel irregularities, generating a more regular and symmetrical surface. A point of debate concerns the unpredictability of the final refractive outcome as the changes induced by the correction are unknown, and a second refractive procedure may be necessary. Laíns et al. evaluated 31 eyes submitted to TG surgery and reported a significant improvement in UDVA and CDVA in the postoperative follow-up. There was a gain of one line or more in uncorrected visual acuity. Furthermore, topographic irregularity in the central 3 mm of the cornea was considerably reduced [62].

Kwitko et al. described a series of cases in which 60 eyes were evaluated in 48 patients. The study included patients with corneal irregularity due to PK, RK, anterior lamellar keratoplasty, and other diagnoses that result in poor visual acuity and dissatisfaction with the quality of vision, all confirmed via topography. That retrospective study reported improved CDVA in 65% of the eyes. A significant reduction in manifest refraction and follow-up confirmed the durability of the results, as in the cases presented here [63].

Some refractive surgeons performed Lasik surgery to correct residual errors, but PRK is most often used due to the possibility of opening the incisions after making the flap [66].

### Cataract surgery and pinhole IOL implants following RK

Some precautions should be considered before cataract surgery. First, the surgeon should examine if the patient had open incisions and good visual acuity (before the development of lens opacity) before the surgery; if this is the case, it would be suitable to consider the solutions presented in this review.

#### IOL Calculation

IOL calculation can be tricky, using IOL calculation software or a more advanced formula is preferable, like Haigis, Hoffer Q, Holladay 2, or SRK/T rather than a regression formula (e.g., SRK I or SRK II) and then choosing the highest resulting IOL power. Keratometric power could be directly measured using corneal topography or scheimplug tomography, applying pre-RK keratometry value minus the refractive change, or adjusting the base curve of a plano contact lens by over-refraction [67–69].

Corneal tomography or scanning-slit interferometry is preferred over corneal topography in patients with a history of RK since it evaluates both anterior and posterior corneal surfaces and has more options to evaluate keratometric values and corneal power. Measurement in the 4-mm paracentral zone of post-RK corneas overestimates corneal power and leads to postoperative hyperopia, so some authors suggest aiming for a myopic correction between -0.50 D and -1.50 D to avoid postoperative hyperopic surprises (some surgeons use this empiric rule a four-cut RK, add 0.5 to 1 D of IOL power an eight-cut RK, add maybe 1 to 1.5 D of IOL power; 12-cut and beyond, add at least 2 D of power). Other authors suggest IOL calculation formulas like Barrett True K, Haigis, and Turnbull in post-RK eyes [67–69].

The True K formula considers the magnitude of RK correction if the data is available. In contrast, the True K [Partial History] and True K [No History] formulas estimate preoperative corneal curvature based on post-RK refraction, biometry, axial length, anterior chamber depth, and current keratometry [67–69].

Savini et al. reviewed IOL calculation after RK. They found that there are limited methods available for determining the corneal power of eyes that have previously undergone RK and are later subjected to phacoemulsification and IOL implantation. The Clinical History Method, which derives keratometric values from refraction and requires knowledge of three critical perioperative data: preoperative keratometric diopters (D), surgically-induced refractive change (SIRC) at the corneal plane, and stabilized postoperative refraction, has traditionally been the method of choice when both pre-and postoperative data are accessible. However, its accuracy may be compromised by hyperopic shifts following RK. Standard keratometric values can often underestimate the corneal power due to alterations in the keratometric index after RK. The radius error of the optical zone can potentially offset this underestimation. To mitigate this, it's recommended to utilize central corneal power measurements, such as the Anterior Central Corneal Power at 3 mm (ACCP3) or the Effective Refractive Power (EffRP) found in the Holladay Diagnostic Summary. Incorporating these measurements into Double-K formulas can lead to precise Effective Lens Position (ELP) predictions. Packer and his team achieved notable results using the EffRP in the Holladay 2 formula, while Awwad et al. reported comparable outcomes with ACCP3 in the Double-K Holladay 1 formula. Furthermore, the IOLMaster's keratometry readings, which capture a smaller diameter, can be integrated into the Haigis formula, sidestepping ELP prediction challenges. Geggel reported positive results using this strategy. There's also a suggestion to use ray tracing to rectify keratometric and radius inaccuracies, but research confirming this approach remains unpublished. Surgeons evaluating IOL power calculations for post-RK eyes must also account for the daily fluctuations in refraction [70].

Wang et al. also studied the same topic and found that eyes that have undergone RK show lower accuracy than those treated with myopic or hyperopic LASIK or PRK. In studies examining the accuracy of newer IOL formulas, results varied: for 95 RK-treated eyes, 29% to 62% were within 0.5 D of target. Potvin and Hill observed a 37% to 47% accuracy rate in 83 eyes. For 44 eyes, both the Barrett True-K No-History and Haigis-TK formulas achieved 43.2% accuracy. Turnbull and colleagues found that the Barrett True-K formulas had 69.2% to 76.6% of eyes within ± 0.5 D of prediction error, while other methods ranged from 40.4% to 69.2%. A study by Awwad reported an 87.5% accuracy rate using average central corneal power over 3.0 mm. The central cornea flattens in eyes treated with myopic LASIK, PRK, or RK, amplifying the positive corneal spherical aberration (SA). The increase in SA correlates with the degree of myopic correction. IOLs with negative SA can help mitigate this effect. Fernández-Vega observed enhanced contrast sensitivity in eyes with multifocal IOLs possessing negative asphericity. Alfonso found aspherical multifocal IOLs provided superior intermediate visual acuity over spherical ones. However, even the most negative SA in IOLs can only partially offset the increased positive ocular SA post-refractive surgery [54].

Patel et al. compared the ASCRS calculator in post-Lasik and RK eyes. They found that using the ASCRS IOL calculator for post-LASIK and post-RK eyes without prior refraction data showed no significant difference in prediction errors (PEs). The 'minimum' IOL power setting yielded the smallest PE variance for post-LASIK eyes, with a higher likelihood of eyes being within ± 1 D post-surgery. For post-RK eyes without prior refraction data, the 'average' IOL power setting was optimal for achieving similar outcomes [71].

Ferrara et al. indicated a marked decrease in ME(mean error) and MAE (mean astigmatic error) using the EVO 2.0, Kane, and PEARL-DGS methods, with no notable differences among them. Their performance improved with 3-mm average keratometry for IOL power calculation, consistent with earlier literature. The Barrett True K formula outperformed the SRK-T, with our data showing an MAE increase from 7.4% (SRK-T) to 25.9% (Barrett True K). EVO 2.0, Kane, and PEARL-DGS showed even higher MAE rates at 59.2%, 74.1%, and 77.8% respectively. The rate of patients with an MAE > 1D dropped from 55.56% (SRK-T) to 0% with EVO 2.0, Kane, and PEARL-DGS. However, while Barrett True K was less effective than the top three formulas, it still showed significant improvement over the SRK-T. Given these results, targeting a ± 0.25 D refractive goal seems prudent, acknowledging the slight hyperopic shift even with the best IOL calculation formulas [72].

#### Intraoperative special care

Placing clear corneal incisions peripherally between two adjacent RK incisions is recommended to minimize the risk of wound dehiscence. The incision size should be 3.2 mm or smaller with eight RK incisions, 2.2 mm or smaller with 12 RK incisions, and 2.0 mm or smaller with 16 RK incisions. A temporal approach is advised with corneal incisions, and using a stabilizing suture as a precautionary measure can help prevent intraoperative wound dehiscence. A study conducted by Meduri et al. on 24 patients with 16 RK incisions found that 37.5% of cases with wound dehiscence had superior incisions without sutures, 20% had temporal incisions without sutures, and none of the cases with temporal incisions and stabilizing suture experienced dehiscence [67–69].

Finally, another safer option is to perform a scleral tunnel, as customary in the 90 s (prior to clear corneal incisions), to avoid intersecting incisions [54, 55, 67–69].

#### Pinhole implants

Recently, IOL pinhole implants in irregular corneas have yielded good results in pseudophakic patients. The same concept can be applied to patients who have undergone RK as well, and some surgeons began using these implants in select cases, as described by Son et al., with good results [56].

### Current application

The corneal incisions still performed are known as Limbal Relaxing Incisions (LRIs). LRIs are nonpiercing, strategically applied incisions that reduce astigmatism before cataract surgery. However, LRIs have been widely used to correct corneal astigmatism. To correct astigmatism, they have been replaced by more precise methods, such as toric intraocular lenses (toric ILI), the application of femtosecond laser during cataract surgery, and the postoperative period of corneal transplantation.

## Conclusion

It is essential to understand the RK procedure, the theoretical background that supported this surgery, the current effect on the cornea, and how to approach patients needing vision improvement. These patients are developing cataracts that need to be handled well, from the IOL calculation to the surgical procedure. Guided keratorefractive surgery is the most accurate procedure to improve these patients' vision and life so far. Nevertheless, some patients may need other approaches, such as sutures, PK, corneal rings, and pinhole implants, depending on the degree of irregularity of the cornea, ablation depth for guided surgery or if the sutures are open.

## Data Availability

Not applicable.
